# Malaria vector control by indoor residual insecticide spraying on the tropical island of Bioko, Equatorial Guinea

**DOI:** 10.1186/1475-2875-6-52

**Published:** 2007-05-02

**Authors:** Brian L Sharp, Frances C Ridl, Dayanandan Govender, Jaime Kuklinski, Immo Kleinschmidt

**Affiliations:** 1 Malaria Research Lead Programme, Medical Research Council, 491 Ridge Rd, Durban, South Africa; 2 One World Development Group, Gainesville, Florida

## Abstract

**Background:**

A comprehensive malaria control intervention was initiated in February 2004 on Bioko Island, Equatorial Guinea. This manuscript reports on the continuous entomological monitoring of the indoor residual spray (IRS) programme during the first two years of its implementation.

**Methods:**

Mosquitoes were captured daily using window traps at 16 sentinel sites and analysed for species identification, sporozoite rates and knockdown resistance *(kdr*) using polymerase chain reaction (PCR) to assess the efficacy of the vector control initiative from December 2003 to December 2005.

**Results:**

A total of 2,807 and 10,293 *Anopheles funestus *and *Anopheles gambiae s.l. *respectively were captured throughout the study period. Both M and S molecular forms of *An. gambiae s.s. *and *Anopheles melas *were identified. Prior to the first round of IRS, sporozoite rates were 6.0, 8.3 and 4.0 for *An. gambiae s.s.*, *An. melas *and *An. funestus *respectively showing *An. melas *to be an important vector in areas in which it occurred. After the third spray round, no infective mosquitoes were identified. After the first spray round using a pyrethroid spray the number of *An. gambiae s.s. *were not reduced due to the presence of the *kdr *gene but *An funestus *and *An. melas *populations declined from 23.5 to 3.1 and 5.3 to 0.8 per trap per 100 nights respectively. After the introduction of a carbamate insecticide in the second round, *An. gambiae s.s. *reduced from 25.5 to 1.9 per trap per 100 nights and *An. funestus *and *An. melas *remained at very low levels. *Kdr *was found only in the M-form of *An. gambiae s.s. *with the highest frequency at Punta Europa (85%).

**Conclusion:**

All three vectors that were responsible for malaria transmission before the start of the intervention were successfully controlled once an effective insecticide was used.

Continuous entomological surveillance including resistance monitoring is of critical importance in any IRS based malaria vector control programme. This paper demonstrates that sufficient resources for such monitoring should be included in any proposal in order to avoid programme failures.

## Background

Malaria is a major endemic disease in the Central African tropical island of Bioko, Equatorial Guinea. High prevalence of infection with malarial parasites in children has been reported [1,2]. *Anopheles gambiae sensu stricto *(*s.s.*), *Anopheles melas *and *Anopheles funestus *are responsible for malaria transmission on the island [3,4] resulting in entomological inoculation rates (EIR) of up to 281 and 787 infective bites per year for *An. gambiae s.s. *and *An. funestus *respectively [4].

Bioko has an all year round humid climate with a short dry season from November to March. The population is estimated at 250,000 most of who live in or around Malabo, the capital of Equatorial Guinea.

In February 2004, The Bioko Island Malaria Control Project (BIMCP) initiated a comprehensive malaria control intervention consisting of indoor residual spraying (IRS) and effective case management, funded by a consortium led by Marathon Oil Company in partnership with the government of Equatorial Guinea, Medical Care Development International (MCDI), One World Development Group (OWDG), Medical Research Council of South Africa (MRC) and Harvard University. The intervention was monitored by entomological, clinical and population indicators [2].

This paper reports on the continuous entomological monitoring of the IRS programme during the first two years of its implementation (2004–2005). Monitoring of the impact of the intervention on prevalence of infection in children has previously been reported [2].

## Materials and methods

### Intervention

The intervention consists of vector control through (IRS) aimed at covering all domicillary structures, and an extensive programme of case management by provision of definitive diagnosis, treatment with artemisinin-based combination therapy, and intermittent preventive treatment (IPT) to pregnant women.

The first round of IRS using the synthetic pyrethoids *Deltamethrin*™ and *Fendona*™ was carried out between March 2004 and August 2004. In 2005, two spray rounds took place, during February-July and August-December respectively, mainly using the carbamate insecticide *Ficam*™. Approximately 100,000 houses were sprayed each round.

### Entomological monitoring

Spray operations were similar to those developed in the Lubombo Spatial Development Initiative (LSDI) in Mozambique, Swaziland and South Africa [5]. Progress and performance of spray operations were continually monitored with the use of a computerized spray management system which was adapted from Booman *et al*. [6].

**Figure 1:**
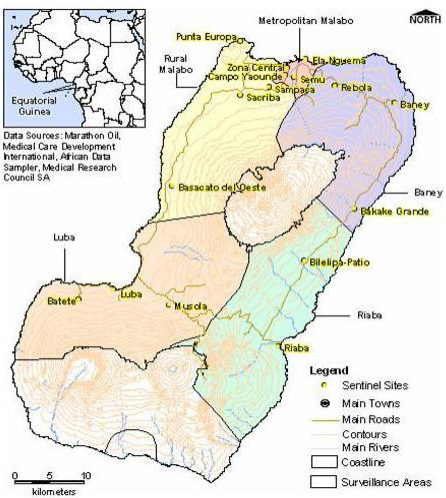
Location of window trap sites used to monitor the Bioko Island Malaria Vector Control Programme intervention.

In November 2003, prior to the implementation of the control programme, window traps were installed at six houses at each of sixteen sentinel sites (Figure 1). Collections were made daily by the trained home owner emptying the contents of the window trap into pre-labeled specimen jars containing isopropanol and completing checklists specifying nights for which traps were operating. The jars were collected and replaced at four week intervals. Mosquitoes were dispatched to the MRC in Durban for morphological identification and molecular analyses.

Mosquitoes were collected prior to the onset of the programme to allow for comparison between pre- and post intervention periods. The number of mosquitoes caught was compared over time, between sentinel sites and with respect to species composition, infection rates and the pattern of pyrethoid knockdown resistance (*kdr*).

### Identification of vector species, sporozoite rates, molecular forms and kdr mutations

Mosquitoes were separated into *Culicinae *and *Anophelinae *and counted. Anophelines were morphologically identified into *An. gambiae s.l. *and *An. funestus *using the keys described by Gillies and De Meillon [7] and Gillies and Coetzee [8] and individually stored in isopropanol for subsequent molecular studies.

DNA was extracted from the head and thorax of a sub-sample of mosquitoes using the Livak method [9]. Members of the *An. gambiae *complex were identified using the Polymerase Chain Reaction (PCR) method described by Scott *et al *[10]. Molecular forms within *An. gambiae s.s *were identified using the protocol of Favia *et al*. [11] and all negative samples or those showing discrepancies were repeated using the protocol of Fanello *et al*. [12]. Members of the *An. funestus *group were identified using PCR as described by Koekemoer *et al*. [13]. The presence of *Plasmodium falciparum *sporozoites was determined using the PCR protocol by Snounou *et al*. [14]. The detection of the west African Leu-Phe *kdr *mutation was based on the PCR method of Martinez-Torres *et al*. [15].

Numbers of mosquitoes per trap per night were calculated for each vector species, both pre- and post-IRS, based on day of capture of the specimen in relation to spray status of the site in which the window trap was located. Using the species specific estimated sporozoite prevalence, the number of infective mosquitoes per trap per night, by species was calculated; the ratio of infective numbers per trap per night post spraying, relative to pre-spraying, was defined as the relative transmission index.

All culicenes caught were recorded to ensure that in the absence of anopheline catches, the traps were being successfully operated.

## Results

### Vector species identification

During this study, which comprised 59,307 trapping nights, 10,293 *An. gambiae s.l. *and 2,807 *An. funestus *were collected and morphologically identified. Of these, 2,043 An. *gambiae s.l. *and 588 *An. funestus *were subjected to species specific analysis (Table 1). *An gambiae s.s. *and *An. melas *were the only two members of the *An. gambiae *complex and *An. funestus *was the only member of the *An*. *funestus *group to be identified. Prior to spraying *An. gambiae s.s. *was identified from all sixteen sentinel sites and *An. melas *from four sites (Basacato del Oeste, Luba, Punta Europa and Riaba). *An. funestus *was identified from ten sites (Basacato del Oeste, Batete, Ela Nguema, Musola, Punta Europa, Rebola, Riaba, Sacriba, Sampaka and Semu). After the third spray round, *An. gambiae s.s. *was identified from eight sites (Baney, Basacato del Oeste, Bilelipa, Campo Yaounde, Luba, Punta Europa, Rebola and Sacriba) and *An. melas *only from Punta Europa. No *An. funestus *were caught in window traps after the third spray round.

**Table 1:** Density, Sporozoite prevalence and Transmission index of specimens collected at sentinel sites in Bioko, 2004–2005

	Pre spray	Post spray 1	Post spray 2	Post spray 3
*An. gambiae s.l.*				
No. caught	2389	7427	341	136
No. analysed for species identification	719	1017	230	77
Proportion *An. gambiae s.s. *(%)	82	97	88	90
				
*An. gambiae s.s.*				
No. estimated	1959	7204	300	122
No. per trap per 100 nights	23.9	25.5	1.9	1.7
Sporozoite rate	6.0(n = 586)	1.4(n = 985)	0(n = 203)	0(n = 69)
Transmission index ^§^	1.4	0.36	0	0
Transmission index relative to baseline	1	0.26	0	0
				
*An. melas*				
No. estimated	430	223	41	14
No. per trap per 100 nights	5.3	0.8	0.3	0.2
Sporozoite rate	8.3(n = 133)	3.1(n = 32)	0(n = 27)	0(n = 8)
Transmission index^§^	0.44	0.03	0	0
Transmission index relative to baseline	1	0.07	0	0
				
*An. funestus*				
No. caught	1920	874	10	3
No. analysed for species identification	372	215	1	0
No. per trap per 100 nights	23.5	3.1	0.06	0.04
Sporozoite rate	4.0(n = 372)	2.3(n = 215	0(n = 1)	*
Transmission index^§^	0.94	0.07	*	*
Transmission index relative to baseline	1	0.08	*	*

* Not computed due to insufficient specimens

§ Number of infective mosquitoes per 100 trap nights

*An. gambiae s.s. *accounted for more than 80% of the *An. gambiae s.l. *captured over the duration of the study period and both the M and the S molecular forms were identified. *An. melas *predominated at one site, Riaba, accounting for 94% of the *An. gambiae s.l. *identified at this site prior to IRS (n = 108). After the third spray round, no *An. melas *were identified from this locality.

### Mosquito densities, sporozoite rates and transmission index

The estimated number of *An. gambiae s.s. *per window trap per 100 nights, pre spraying was >20 with *A. melas *markedly lower at <5. *An. gambiae s.s. *showed an increase in the estimated number per window trap post spray round 1 (from 23.9 to 25.5 per trap per 100 nights) but was markedly reduced to two mosquitoes per trap per 100 nights after the second spray round with carbamate insecticide (Table 1). Both *An. melas *and *An. funestus *showed significant reductions after the first spray round (from 5.3 to 0.8 and from 23.5 to 3.1 respectively) and continued to drop further with the subsequent spray round (to 0.2 and 0.04 respectively).

Prior to the onset of IRS, large variations in mosquito numbers existed between sentinel sites with densities of *An. gambiae s.l. *and *An. funestus *ranging from <1 per trap per 100 nights to 182.9 (Punta Europa) and 140.9 (Sacriba) per trap per 100 nights respectively.

Sporozoite prevalence prior to spraying was 6.0%, 8.3%, and 4.0% for *An. gambiae s.s.*, *An. melas *and *An. funestus *respectively. After the first spray round with a pyrethoid spray, these reduced to 1.8%, 3.1% and 2.3% and after the second spray round, no infective mosquitoes were identified (Table 1).

The relative transmission index (the number of infective mosquitoes per trap per night relative to pre-intervention) was 0.26, 0.07, 0.08 for *An. gambiae s.s.*, *An. melas *and *An. funestus *respectively after the first spray round. All mosquitoes tested after the second spray round were negative for sporozoites so no transmission index could be calculated (Table 1).

### Culicene densities

Prior to the onset of IRS, *culicene *densities were 130.2 per trap per 100 nights. Post spraying, densities were 108.0, 115.6 and 81.4 per trap per 100 nights for each spray round respectively. Large variations in *culicene *numbers existed between sentinel sites, throughout the study period with densities ranging from 6 to 461 per trap per 100 nights.

### M and S molecular forms of *An. gambiae s.s. *and the relationship to kdr

Prior to the onset of spraying, 64% of *An*. *gambiae s.s. *were identified as S-forms and 36% as M-forms (n = 362). The proportion of M forms rose steadily with each spray round and by the end of the third spray round, 80% of *An. gambiae s.s. *were identified as M forms (n = 63). S-forms only were found at Baney, Batete, Bilelipa, Musola and Rebola and M-forms only from Central and Bacake Grande. M and S forms were sympatric at all other localities. No hybrids were identified.

Prior to the onset of IRS, 50% of M-forms had either the homozygous resistant (*kdr/kdr*) or the heterozygous susceptible (*kdr/+*) *kdr *genotypes (Table 2). By the end of the third spray round, 78% of M-forms had either one of the *kdr *genotypes. *Kdr *was absent in all S-forms. The *kdr *mutation was identified at eight sentinel sites, Basacato del Oeste, Punta Europa, Sampaka, Sacriba, Campo Yaounde, Central and Semu, all of which are in the north-west of the island and Luba in the south-west.

**Table 2:** Kdr in M-forms of *An. gambiae s.s. *collected in Bioko, 2004–2005

	Total number tested	Number Resistant (kdr/kdr)	Number Susceptible/Resistant (+/kdr)	Number Susceptible (+/+)	Percent Resistant (R and SR)
Pre spray	105	16	37	52	50
Post spray 1	132	22	61	49	63
Post spray 2	102	27	42	33	68
Post spray 3	30	4	19	7	78

## Discussion

Prior to intervention by IRS, *An. gambiae s.s. *and *An. funestus *were shown to be the major malaria vector species biting on the island of Bioko. This corroborates previous reports of the anopheline distribution and their infectivity for the island [4,16]. The first round of IRS with a pyrethoid insecticide dramatically reduced the *An. funestus *and *An. melas *populations which remained at very low levels following second and third round IRS with a carbamate. The first IRS round with a pyrethoid had no effect on the number of *An. gambiae s.s. *collected, but did reduce their sporozoite rate, thereby substantially lowering their transmission potential as expressed by the transmission index. This is likely to be the result of a change in the age structure of *An. gambiae s.s. *after the first round of spraying with a pyrethoid. It was only after the introduction of a carbamate insecticide that the number of *An. gambiae s.s. *exiting through window traps were significantly reduced and remained low with subsequent IRS rounds with a carbamate.

*An. melas *was implicated as an important vector of malaria in localized areas in which it occured. Its distribution was restricted to coastal areas and had the highest infectivity rate of the three species prior to IRS intervention and after the first spray round. The transmission index was however lower due to the low numbers caught. Akogbero *et al*. [17] found that the infectivity of *An. melas *in coastal areas of Benin was lower compared to that of *An. gambiae s.s. *in places where *An. melas *was most abundant. Morreno *et al*. [18], found similar infection rates for both *An. melas *and *An. gambiae s.s. *in Equatorial Guinea (mainland). The present findings corroborate this study and show that in areas where both vectors cohabit (Punta Europa and Riaba), regardless of which one is dominant, infectivity rates were similar.

The two genetic variants of *An. gambiae s.s.*, the molecular M form and S form were both identified throughout the study period. The data indicated a strong selection for the M form over the IRS intervention period, which is linked to the presence of the *kdr *mutation.

**Figure 2:**
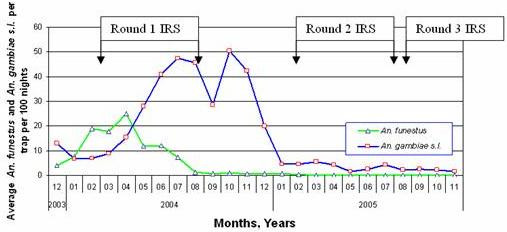
Average number of *An. gambiae s.l. *and *An. funestus *per window trap per 100 nights, Bioko, December 2003–November 2005.

A study on Bioko between 1998 and 2001 reported no evidence of *kdr *in the *An. gambiae s.s. *population despite the use of pyrethoid-impregnated bednets [3]. It was on the basis of this study that the decision was made to implement IRS with a pyrethoid insecticide. However, retrospective analysis of samples collected prior to IRS and analysed in 2004, showed the presence of *kdr *in 50% of M-forms tested but absent in the S form (Table 2). This was confirmed by Reimer *et al*. [19] who reported the *kdr *gene present in 55% of molecular M-forms of *An gambiae s.s. *from Malabo but absent from the S-form. The frequency of the *kdr *allele continued to rise and by the end of 2005, was present in 78% of M-forms, but absent in S-forms and reached a maximum frequency of 85% at Punta Europa sentinel site. Pyrethroids were mainly withdrawn from use in IRS in 2004 after the first spray round and replaced with a carbamate insecticide. However, pyrethroids were still frequently used in the form of vehicle and hand fogging in Malabo [19]. Some pyrethroids were also used in the IRS programme as late as 2005 constituting 17% of the total structures sprayed. Thus pyrethroids from fogging, IRS and ITNs were present for selection resulting in the increasing frequency of the M form of *An. gambiae s.s. *due to the presence of the *kdr *gene. Figure 2 shows the continual increase in *An. gambiae s.l. *caught exiting houses from March 2004 in spite of the implementation of the IRS programme using a pyrethroid. The failure to control *An. gambiae s.s. *effectively with a pyrethroid due to *kdr *is demonstrated by the effective reduction in the seasonal peak in *An. gambiae s.s. *after the introduction of carbamate IRS (Figure 2). In contrast to *An. gambiae s.s. *the relative abundance of house leaving *An. funestus *and *An. melas *was dramatically reduced following the first round of IRS using a pyrethroid and remained low with the introduction of a carbamate. The efficacy of a carbamate insecticide for large scale IRS was recently also demonstrated in Mozambique for both *An. arabiensis *and non *kdr *pyrethroid resistant *An. funestus *[5].

This study further emphasizes the importance of monitoring and evaluation in any large scale insecticide based vector control programme. The efficacy of the IRS programme in Bioko is demonstrated by the dramatic reduction in *An. funestus *and *An. melas *in the first spray round and *An. gambiae s.s. *by the second spray round, and the substantial decrease in the transmission index after the first year, a finding supported by the significant decrease in the parasite prevalence in children by one third after the first year of IRS [2].

These results lead us to emphasise the critical importance of continuous entomological surveillance including insecticide resistance monitoring based on an extensive system of window traps in any IRS based malaria vector control programme. It is recommended that proposals for such interventions include sufficient resources for such monitoring to avoid programme failures that may result from the ineffective control of important malaria vectors.

## Authors' contributions

BLS: Co-designed the study, participated in analysis and interpretation of data and contributed to the drafting of the manuscript. FCR carried out the laboratory analyses of mosquitoes, assisted with data analysis and interpretation and was involved in the drafting of the manuscript. DG: Managed the database, assisted with the analysis of results and contributed to the manuscript. JK: Management of IRS and the collection of field samples and contributed to the manuscript. IK: Co-designed and coordinated the study, participated in the analysis and interpretation of results, and was involved it the drafting of the manuscript and critical evaluation thereof.

All authors read and approved the manuscript.

## References

[B1] Roche J, Ayecaba S, Amela C, Alvar J, Benito A (1996). Epidemiological characteristics of malaria in Equatorial Guinea. Res Rev Parasitol.

[B2] Kleinschmidt I, Sharp B, Benevente L, Schwabe C, Torrez M, Kuklinski J, Morris N, Raman J, Carter J (2006). Reduction in infection with *Plasmodium falciparum *one year after the introduction of malaria control interventions on Bioko Island, Equatorial Guinea. Am J Trop Med Hyg.

[B3] Berzosa PJ, Cano J, Roche J, Rubia JM, Garcia L, Moyano E, Guerra A, Mateos JC, Petraca V, Do Rosaria V, Benito A (2002). Malaria vectors in Bioko island (Equatorial Guinea): PCR determination of the members of *Anopheles gambiae *Giles complex (Diptera: Culicidae) and pyrethoid knockdown resistance (*kdr*) in *An. gambiae *sensu stricto. J Vector Ecology.

[B4] Cano J, Berzosa PJ, Roche J, Rubio JM, Moyano E, Guerra-Neira H, Brochero H, Mico M, Edu M, Benito A (2004). Malaria vectors in the Bioko Island (Equatorial Guinea): estimation of vector dynamics and transmission intensities. J Med Entomol.

[B5] Sharp BL, Kleinschmidt I, Streat E, Maharaj R, Barnes KI, Durrheim DN, Ridl FC, Morris N, Seocharan I, Kunene S, La Grange JJP, Mthembu DJ, Maartens F, Martin CL, Barreto A (2007). Seven years of regional malaria control collaboration-Mozambique, South Africa and Swaziland. Am J Trop Med Hyg.

[B6] Booman M, Sharp BL, Martin CL, Manjate B, La Granga K, Durheim DN (2003). Enhancing malaria controlusing a computerized management system in Southern Africa. Malar J.

[B7] Gillies MT, De Meillon B (1968). The Anophelinae of Africa south of the Sahara.

[B8] Gillies MT, Coetzee M (1987). A Supplement to the Anophelinae of Africa South of the Sahara (Afrotropical Region).

[B9] Collins FH, Mendez MZ, Rasmussen MO, Mehaffey PC, Bensansky NJ, Finnerty V (1987). A ribosomal RNA gene probe differentiates member species of the *Anopheles gambiae *complex. Am J Trop Med Hyg.

[B10] Scott JA, Brogdon WG, Collins FH (1993). Identification of single specimens of the *Anopheles gambiae *group by polymerase chain reaction. Am J Trop Med Hyg.

[B11] Favia G, Lanfracotti A, Spanos L, Siden-Kiamos, Louis C (2001). Molecular characterization of ribosomal DNA polymorphisms discriminating among chromosomal forms of *Anopheles gambiae s.s.*. Insect Mol Biol.

[B12] Fanello C, Santolamazza F, della Torre A (2002). Simultaneous identification of species and molecular forms of the *Anopheles gambiae *complex by PCR-RFLP. Med Vet Entomol.

[B13] Koekemoer LL, Kamau L, Hunt RH, Coetzee M (2002). A cocktail polymerase chain reaction assay to identify members of the *Anopheles funestus *(Diptera: Culicidae) group. Am J Trop Med Hyg.

[B14] Snounou G, Viriyakosol S, Zhu XP, Jarra W, Pinheiro L, do Rosario VE, Thaithong S, Brown KN (1993). High sensitivity of detection of human malaria parasites by the use of nested polymerase chain reaction. Mol Biochem Parasitol.

[B15] Martinez-Torres D, Chandre F, Williamson MS, Darriet F, Berge JB, Devenshire AL, Guillet P, Pasteur N, Pauron D (1998). Molecular characterization of pyrethoid knockdown resistance (*kdr*) in the major malaria vector *Anopheles gambiae s.s.*. Insect Mol Bio.

[B16] Molina R, Benito A, Roche J, Blanca F, Amele C, Sanchez A, Alvar J (1993). Baseline entomological data for a pilot malaria control programme in Equatorial Guinea. J Med Entomol.

[B17] Akogbero M, Romano R (1999). Infectivity of *Anopheles melas *vis-à-vis *Plasmodium falciparum *in the coastal lagoon area of Benin. Bull Soc Pathol Exot.

[B18] Moreno M, Cano J, Nzambo S, Bobuakasi L, Buatiche JN, Ondo M, Micha F, Benito A (2004). Malaria Panel assay vs PCR: detection of naturally infected Anopheles melas in a coastal village of Equatorial Guinea. Malar J.

[B19] Reimer LJ, Tripet F, Slotman M, Spielman A, Fonjo E, Lanzaro GC (2005). An unusual distribution of the *kdr *gene among populations of *Anopheles gambiae *on the island of Bioko, Equatorial Guinea. Insect Mol Biol.

